# How did beliefs and perceptions about e-cigarettes change after national news coverage of the EVALI outbreak?

**DOI:** 10.1371/journal.pone.0250908

**Published:** 2021-04-30

**Authors:** Jennifer C. Morgan, Nathan Silver, Joseph N. Cappella

**Affiliations:** Annenberg School for Communication, University of Pennsylvania, Philadelphia, PA, United States of America; Medical University of South Carolina, UNITED STATES

## Abstract

**Introduction:**

Exposure to media content can shape public opinions about tobacco. In early September 2019, the outbreak of e-cigarette, or vaping, product use–associated lung injury (EVALI) became headline news in the United States.

**Methods:**

In August and September 2019, we conducted two cross-sectional online surveys with current and former smokers assessing attitudes and beliefs about e-cigarettes. Study one (*n* = 865) was collected before the EVALI outbreak was widely covered and study two (*n* = 344) was collected after the outbreak had become nation-wide news. We examined differences in perceptions and beliefs between time points.

**Results:**

E-cigarette harm perceptions increased between study one (mean = 2.67) and study two (mean = 2.90, p < .05). Ever-users of e-cigarettes largely account for this change. Endorsement of the belief that e-cigarettes were risky and more likely to cause lung damage compared to cigarettes increased between studies (p < .05). Seventy eight percent of participants at study two were aware of the vaping illness story. Being aware of the story was associated with more endorsement of the belief that e-cigarettes were risky to use, but not that using e-cigarettes would make the participant more likely to get damaged lungs.

**Discussion:**

When the stories about the health and safety of tobacco products dominate the public information environment, it presents an opportunity to change beliefs that are frequently targeted by paid health campaigns. Changes in participant’s perceptions of e-cigarettes were associated with coverage of this large news story, underscoring the importance of working to ensure that coverage is a scientifically accurate as possible.

## Introduction

Electronic cigarettes, or vaping devices, produce an inhalable aerosol that usually contains nicotine, flavorings and other chemicals [1]. While the name “vaping” conjures the idea of harmless water vaper, the aerosol can expose users to heavy metals, volatile organic compounds and other harmful ingredients know to have adverse health effects [2, 3]. In August 2019, Wisconsin reported the first cluster of lung injuries to the Centers for Disease Control and Prevention (CDC) [4]. The CDC worked with federal and state partners to address a multistate outbreak of e-cigarette, or vaping, product use–associated lung injury (EVALI) [5]. A total of 2807 cases of EVALI and 68 deaths had been reported to the CDC as of February, 2020 [5]. All patients with EVALI have reported using vaping products. Nationally, most of these patients reported using *Tetrahydrocannabinol* (THC)-containing products, but a minority reported exclusive use of nicotine-containing products [5, 6].

Beginning in September 2019, news surrounding a vaping illness simultaneously prompted restrictions and bans by local governments because of the questions about the safety of electronic nicotine delivery systems [6], *and* a defense of e-cigarettes by industry representatives claiming that it was bootleg, cannabis-based cartridges that were leading to the injuries, and not the nicotine-based cartridges that were sold legally [7]. Extensive coverage of EVALI was likely effective in spotlighting the issue of e-cigarettes safety [8]. However, conflicting information about the cause, and uncertainty about the cause when the news first broke [7–9], may have limited the ability of the news to perform one of its functions and accurately inform people about the actual and potential dangers [10–12].

The U.S. Food and Drug Administration (FDA) acknowledges that in comparison to combustible cigarettes, which kill up to half of life-long smokers, e-cigarettes lie on the lower end of a continuum of risk [13–15]. Yet, many smokers in the U.S. believe e-cigarettes are at least as harmful to health as combustible cigarettes [16]. This may dissuade them from switching to e-cigarettes and, thus, have a detrimental impact on population health. Widespread news coverage of the EVALI outbreak may have increased confusion about the relative harms of these products.

Media scholars posit that mass media serves four core functions: (*a*) information distribution, reporting events to the public; (*b*) interpretation, providing the context for and meaning of issues and events; (*c*) socialization, cultivating community values, beliefs, and norms; and (*d*) entertainment, providing diversion and escape from everyday life [17, 18]. Though news media can affect policy and behavior change through providing a source of health and science information [19–21], the effect of that information is not consistent. Thus, we sought to examine whether the *initial* widespread news coverage of EVALI changed perceptions and beliefs about e-cigarettes, especially beliefs about the harms and risks of e-cigarettes among users.

## Methods

### Overview

**Fig 1:**
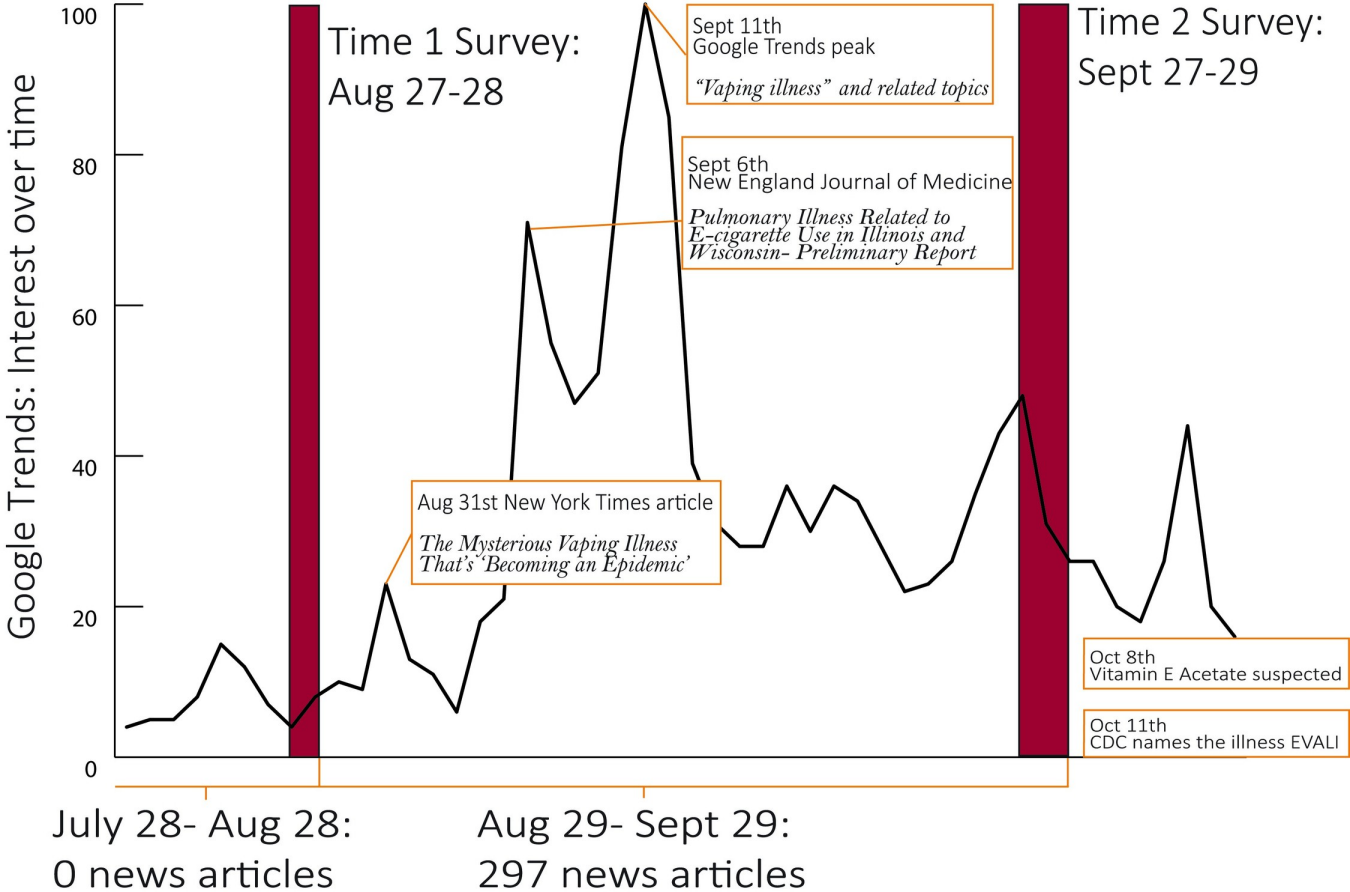
EVALI news coverage in Fall, 2019.

This paper combines the results from two studies to provide data on our research questions. Study one was conducted to assess awareness, harm perceptions and beliefs about three different tobacco products: e-cigarettes, snus, and heat-not-burn tobacco, that have the potential to be authorized as modified risk tobacco products (MRTPs) [22]. This study was completed before EVALI news coverage. Within a week of concluding data gathering for study one, news coverage of EVALI increased substantially. A month before our first study (July 28th to Aug 28th), a ProQuest search in the recent news database using the terms “vaping illness” OR “e-cigarette illness” OR “mysterious lung disease” OR “lung illness” yields 0 news stories. From Aug 29^th^- Sept 29^th^, the same search yields 297 entries, including a front-page New York Times article (Fig 1). Given this opportunity for a natural experiment, we went back into the field with the same measures in order to assess how perceptions and beliefs about e-cigarettes had changed after the news coverage (study two). Like study one, study two survey also assessed awareness, harm perceptions, and beliefs about e-cigarettes. We added items to study two survey that assessed awareness of the news story. Study two was conducted before the “mysterious vaping illness” was given the name EVALI, and before THC or vitamin E acetate was widely accepted as the cause.

### Participants

#### Study one

We recruited 865 adult current and former smokers to complete an online survey about MRTP beliefs through Dynata from August 27–28, 2019. Using established definitions of smoking status [23], participants were considered current smokers if they had smoked at least 100 cigarettes in their lifetime and currently smoke every day or some days, and former smokers if they had smoked at least 100 cigarettes in their lifetime, and currently did not smoke at all. Additionally, participants could not have participated in more than two online surveys about cigarette smoking or other tobacco products in the last three months. Participants included 450 men and 414 women, with a mean age of 47.5 years. A little more than half of the participants were current smokers or ever-users of e-cigarettes. Participants had similar demographics to a national sample of smokers [23], as they were diverse in race, education, and income, though our sample was slightly more educated than the population of US smokers. The purpose of study one was to understand what people believe about MRTPs, and how information they get from brands might influences those beliefs. The sample size for study one allows for comparisons between the three products within subgroups of smoker and non-smoker, and between groups with differing experiences with MTRPs.

#### Study two

Using Dynata and the same inclusion and exclusion criteria, we recruited an independent sample of 344 adult current and former smokers from September 27–29, 2019. Participants included 163 men and 181 women, with a mean age of 46.7 years. Participant characteristics were statistically similar between the two studies (Table 1). The sample size for study was chosen to allow for meaningful comparisons between subgroups of e-cigarette users and between time periods.

**Table 1:** Number and percentage of participants with various demographic characteristics.

Characteristic	Study 1 (n = 865)	Study 2 (n = 344)
	*n (*%)	*n (*%)
**Mean age (SD)**	47.5 (17.6)	46.7 (16.4)
**Gender**		
**Male**	450 (52)	163 (47)
**Female**	414 (48)	181 (53)
**Hispanic**	59 (7)	37 (11)*
**Race**		
**White**	732 (85)	296 (86)
**Black or African American**	81 (9)	18 (5)
**Asian**	14 (2)	1 (.3)
**American Indian, Alaska Native, or Native Hawaiian**	34 (4)	20 (6)
**Multiple races selected**	--	9(3)
**Education**		
**High school or less**	283 (33)	134 (39)
**Some college**	201 (23)	64 (19)
**College or higher**	380 (44)	146 (42)
**Income**		
**Less than $25,000**	187 (22)	79 (23)
**Between $25,000 and $49,999**	213 (25)	83 (24)
**Between $50,000 and $74,999**	164 (19)	66 (19)
**Between $75,000 and $99,999**	126 (15)	53 (15)
**Between $100,000 and $149,999**	117 (14)	35 (10)
**$150,000 or more**	55 (6)	27 (8)
**Smoking status**		
**Current smoker**	463 (54)	201 (58)
**Former smoker**	402 (46)	143 (42)
**E-cigarette user status**		
**Current**	284 (33)	116 (34)
**Former**	168 (19)	68 (20)
**Never**	412 (48)	160(47)

Note. Pearson *X*^2^ test

*p < .05. Missing data ranged from 0% to .5%

### Procedures

#### Study one

Eligible participants answered demographic survey questions and information about their current smoking behavior. Study one’s purpose was to understand what people believe about MRTPs, and how information they get from brands might influences those beliefs. Participants read a paragraph describing the potential for the FDA to authorize MRTPs and a brief description of MRTPs. Participants were randomly assigned to either the control condition, in which they read a generic description about e-cigarettes, snus, and heat-not-burn tobacco, presented in random order; or to the corporate social responsibility condition, in which they read the generic description, plus a corporate responsibility statement crafted using press releases and text from IQOS, General Snus, or JUUL’s website respectively. There were no differences between the conditions, so the conditions were collapsed in subsequent analyses.

After reading descriptions of MRTPs, and a description of the products, participants answered questions about awareness, harm perceptions, use, and susceptibility to each of the products. Then the survey assessed 15 different beliefs for the three products. The belief questions were worded using three different variations and participants were randomly assigned one wording variation per product using a Latin-square design.

Participants read a consent form that provided the approximate time it would take to complete the survey, emphasized that their participation was voluntary, and supplied the contact information for the study PIs and the Institutional Review Board. After reading through the consent form, they were instructed to click through to the next page. The consent form advised those who did not wished to participate to close their internet browsers. The University of Pennsylvania institutional review board approved the consent process, study procedures, and study materials.

#### Study two

Study two was designed to take advantage of the fact that study one had occurred just prior to a large national news story about one of the products. Participants in study two completed a very similar survey. The survey was identical up until the Latin-square design assessment of the 15 beliefs. Study two only assessed beliefs about e-cigarettes. Measures assessing awareness of health news stories from the previous two months were added after the belief assessment in study two.

### Measures in study one and study two

#### E-cigarette use

Participants who answered that they had heard of e-cigarettes before the time of the survey indicated whether they had ever used an e-cigarette or vaping device, even one or two times. Participants who answered yes responded with how many of the last 30 days they had used the device(s). Participants who were unaware of e-cigarettes, or who had never used an e-cigarette were considered never users. Participants who answered that they had used an e-cigarette but reported that they had not used one in the last 30 days were considered former users. Those who had used an e-cigarette at least once in the last 30 days were considered current users of e-cigarettes. Because the participants were all current or former smokers, the former and current users have experience with both cigarettes and e-cigarettes.

#### Harm perceptions

Participants indicated how harmful they thought e-cigarettes were to their health on a scale of not at all harmful (1), to extremely harmful (4). This measure was adapted from the Population Assessment of Tobacco and Health survey [24].

#### E-cigarette beliefs

The surveys assessed 15 different beliefs about e-cigarettes: being risky, having long term health benefits, causing lung damage, tasting good, feeling harsh, being odorless, being easy to use, looking cool, making second hand smoke, not being addictive, containing nicotine, helping smokers quit, untrustworthy science about the product, appealing to kids, and being expensive (Appendix A). The decision to focus on these beliefs was based on prior qualitative research, surveys on salient beliefs, and examining the MRTP applications and materials made publicly available [22, 25–34]. The 5-point response scale ranged from strongly disagree (1) to strongly agree (5). The survey assessed these beliefs using three wording variations in study one: a comparison to cigarettes, a self-referent, and an absolute statement. Spearman ranked correlations of the beliefs between the absolute wording belief ranking and the self-referent wording ranking was very high (ρ (rho) = .98, p < .001) indicating that participants did not meaningfully differentiate the two wording variations [35]. Consequently, in study two, only the comparison and self-referent wording variations were used (ρ (rho) = .63, p < .05).

### Measure in study two

#### Awareness of EVALI story

The survey presented the participants with five one-sentence descriptions of news stories about health that had been run in the past two months. These five stories were presented in a random order and included “Several patients around the country have died because of a mysterious lung ailment tied to vaping.” Other story options were about cardiovascular health of dog owners, allergic reactions because of tattoos, pharmaceutical companies facing fines because of their ties to opioids, and the percent of American youth who had tried vaping. Participants indicated whether they had heard of, read, or seen the story (coded as 1) or not (coded as 0), or if they didn’t know (coded as 0).

### Statistical analysis

The research questions motivating the analysis were:

1. Did harm perceptions and beliefs about e-cigarettes change between study one and study two, particularly for beliefs about health harms or benefits?
2. Did changes between study one and study two differ by e-cigarette user status?

We conducted t-tests adjusted for unequal sample sizes and *X*^2^ in STATA 14.0 [36] to examine differences in harm perceptions between time points. To prevent multiple comparisons from increasing the false positive rates, we conducted a MANOVA for beliefs, followed by post hoc analysis when the MANOVA indicated significant differences between study one and study two. The University of Pennsylvania institutional review board approved the procedures.

## Results

### News coverage and perceived harm

Between study one and study two, during which news coverage of EVALI was intense relative to baseline (Fig 1), the information circulating in the public information environment was associated with an increase in e-cigarette harm perceptions (mean = 2.67 (sd = .90) to m = 2.90 (.97), p < .001). This change was largely driven by ever-users of e-cigarettes, specifically former-users (m = 2.61 (.81) to m = 2.99(.94,) p < .01). Never users’ perceptions trended in the same with a smaller and non-significant difference between time 1 and 2 (m = 2.98(.88) to m = 3.14(.92), p = .051; Table 2).

**Table 2:** E-cigarette harm perception changes between study one and two for never, former, and current users of e-cigarettes.

	Study one Mean (SD)	Study two Mean (SD)	Effect size Hedges’ g
All participants	2.67 (.90)	2.90 (.97)*	-.25
Never user	2.98 (.88)	3.14 (.92)	-.18
Former user	2.61(.81)	2.99 (.94)*	-.43
Current user	2.27 (.81)	2.52 (.94)*	-.29

Note: E-cigarette health harm perceptions were measured on a scale of not at all harmful (coded as 1) to extremely harmful (coded as 4).

*p < .05

### Fifteen beliefs about e-cigarettes

**Fig 2:**
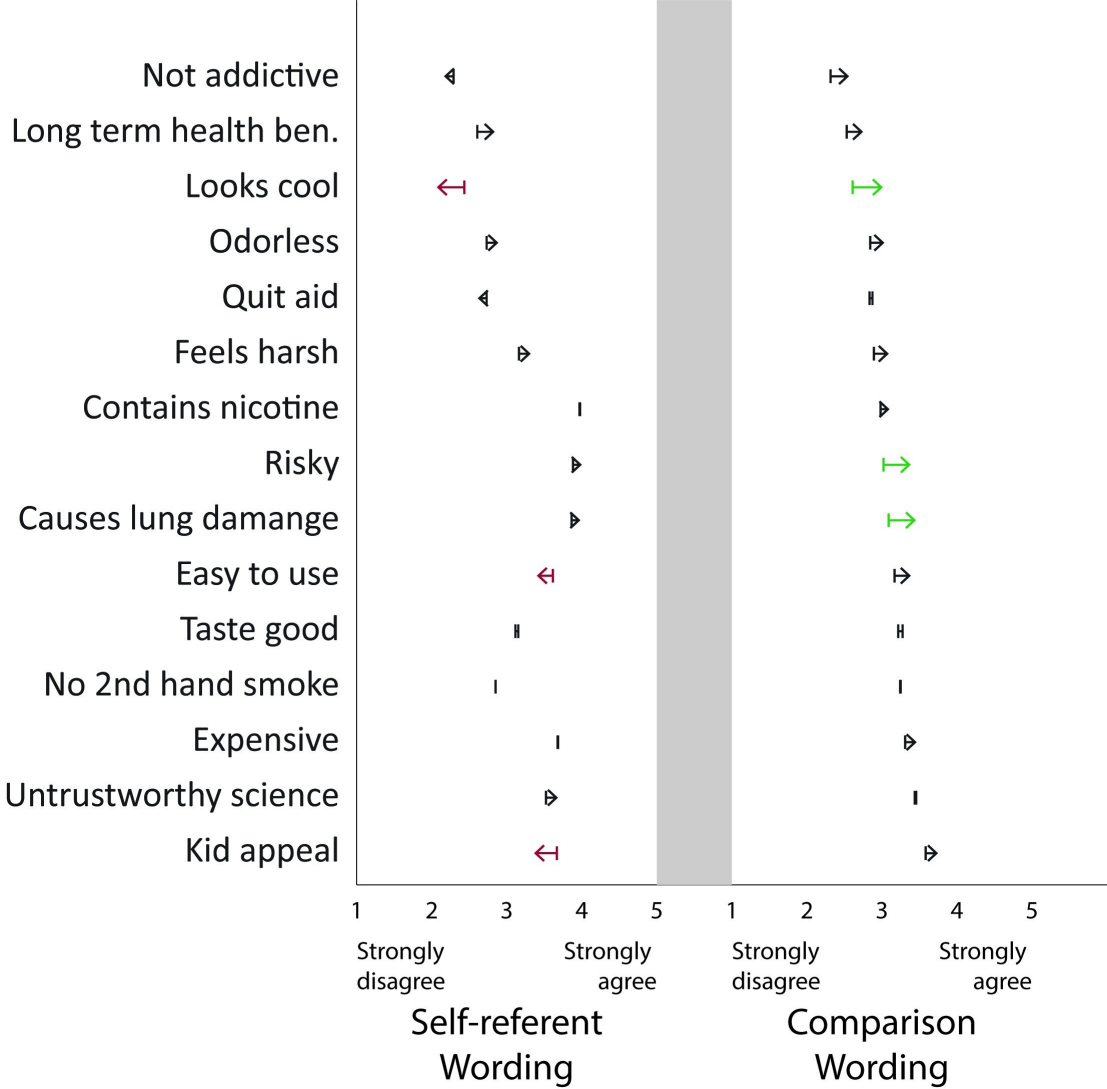
E-cigarette belief changes from study one to study two. A MANOVA indicated significant differences in beliefs from study one and study two. Bold arrows indicate the beliefs that were significantly different between study one and study two (p < .05). The grey bar separates the wording variations.

Between study one and study two some important beliefs did not change significantly after intense news coverage of EVALI; the risk of e-cigarette use, lung damage and long-term health benefits. Some social beliefs about e-cigarettes being easy to use, cool, and appealing to kids decreased between study one and two by .2 to .4 points (p < .05; Fig 2). When *the comparison to cigarettes* was invoked, the belief that e-cigarettes were riskier, more likely to cause lung damage, and cooler increased by .3 to .4 points between study one and two (p < .05). Increased information in the public information environment about e-cigs is affecting beliefs about cigarettes, but only when a comparison to cigarettes is invoked.

A MANOVA did not indicate significant differences between study one and study two for the set of 15 beliefs for current users, or when a comparison to cigarettes was invoked. A MANOVA indicated significant differences between study one and study two for the set of 15 beliefs for never and former users, therefore we conducted post hoc t-tests to examine which of the 15 beliefs changed significantly between study one and study two. *For current users*, this indicates that there was no significant difference in the endorsement of beliefs about e-cigarettes being risky, having long term health benefits, or causing lung damage between study one and study two. In comparison, *never user’s* beliefs about e-cigarettes causing lung damage, being risky to use, and feeling harsh, increased after EVALI news coverage (p < .05; Table 3). The group most at-risk (current users) did not accept the lung damage risk assessment, while the group at lowest risk (never users) did. Information in the public information environment about e-cigarettes is not affecting the at-risk current user group.

**Table 3:** E-cigarette belief change differences over time by user status.

	Self-referent						Comparison					
	Never		Former		Current		Never		Former		Current	
	Study 1	Study 2	Study 1	Study 2	Study 1	Study 2	Study 1	Study 2	Study 1	Study 2	Study 1	Study 2
**Risky**	4.08	4.36*	3.97	4.24	3.57	3.31	3.24	3.49	3.11	3.35	2.58	3.12
**Lung damage**	4.03	4.29*	3.92	4.00	3.61	3.48	3.27	3.58	2.98	3.26	2.87	3.30
**Long-term health benefits**	2.36	2.53	2.50	2.78	2.89	3.07	2.44	2.31	2.32	2.84	2.73	3.18
**Cool**	2.07	1.70*	2.21	1.57*	2.93	2.78	2.36	2.60	2.30	3.06	3.10	3.44
**Untrustworthy sci**	3.68	3.86	3.53	3.97*	3.24	3.28	3.76	3.54	3.25	3.58	3.04	3.25
**Feel harsh**	3.27	3.55*	3.16	3.32	2.94	2.88	3.09	3.06	2.69	2.71	2.68	3.21
**Easy**	3.36	3.01*	3.71	3.57	3.91	3.86	2.88	2.99	3.04	3.42	3.68	3.84
**Taste good**	2.70	2.57	3.14	2.81	3.61	3.83	2.91	2.85	3.00	3.48	3.80	3.75
**No second-hand smoke**	2.51	2.42	2.95	2.49	3.12	3.41	2.94	2.95	3.25	3.39	3.67	3.60
**Odorless**	2.53	2.56	2.70	2.62	2.91	3.29	2.72	2.72	2.58	2.74	3.17	3.53
**Nicotine**	3.95	3.96	4.16	4.24	3.93	3.86	3.06	3.13	2.96	2.68	2.81	3.14
**Quit aid**	2.28	2.18	2.59	2.16	3.34	3.45	2.53	2.34	2.63	2.84	3.52	3.53
**Not addictive**	2.12	1.90	1.98	1.73	2.53	2.69	2.04	2.22	1.91	2.16	2.93	3.14
**Kid appeal**	3.81	3.61	3.53	3.22	3.58	3.09	3.63	3.77	3.36	3.65	3.62	3.79
**Expensive**	3.88	4.00	3.73	3.54	3.43	3.36	3.43	3.40	3.20	3.52	3.13	3.53

Note. E-cigarette beliefs were measured on a scale of strongly disagree (coded as 1) to strongly agree (coded as 5). The MANOVA did not indicate significant differences between study one and study two for the set of 15 beliefs for current users, or anyone who answered the comparison wording variation (greyed out cells), therefore we did not conduct post-hoc analyses examining differences for individual beliefs in those sub groups. Significant differences in post hoc t-tests on individual beliefs (white cells) are indicated with an * indicating a p < .05.

### Awareness of coverage

**Fig 3:**
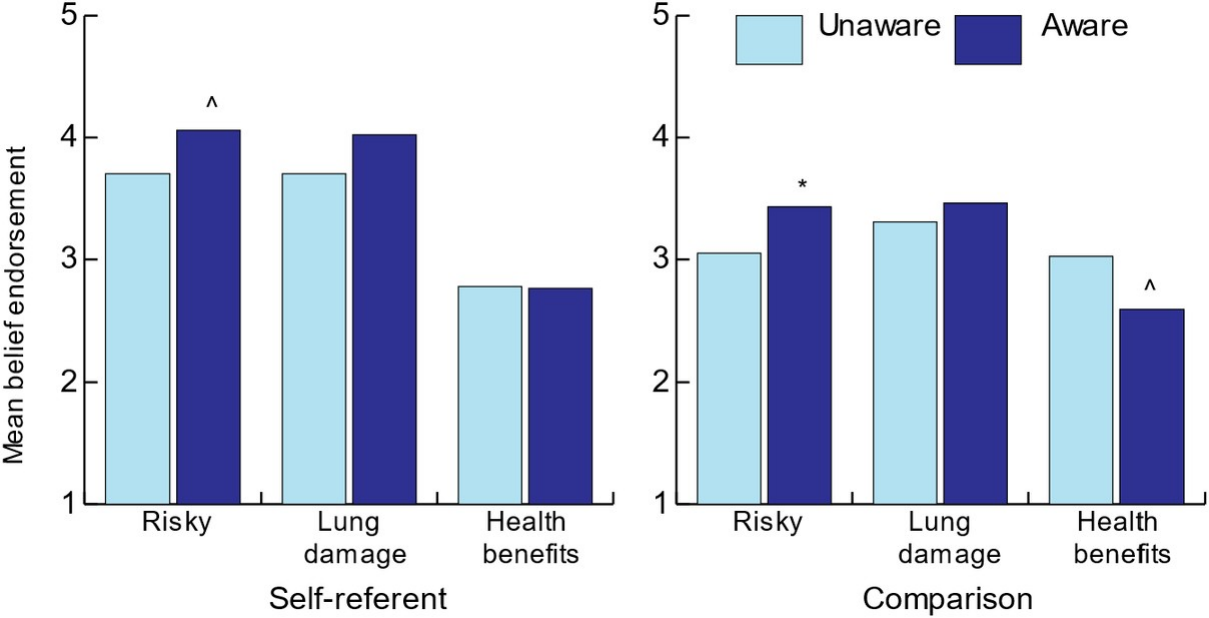
Mean belief endorsement differences in study two among participants who were unaware of the EVALI news compared to those who were aware. Number of unaware participants = 76 (n = 37 for self-referent wording and n = 39 for comparison wording). Number of aware participants = 276 (n = 135 for self-referent and n = 132 for comparison wording). Welch t-test implemented to account for unequal sample sizes, *p < .05 ^p = .07.

Seventy eight percent of participants in study two were aware of the EVALI story, the highest of any of the stories we asked about. In comparison, 22% of people had heard about allergic reactions because of tattoos, 36% about the cardiovascular health of dog owners, 56% about the percent of American youth who had tried vaping, and 57% about pharmaceutical companies facing fines because of their ties to opioids. Those aware of the EVALI story were more likely to endorse the belief that e-cigarettes were risky compared to cigarettes (m_unaware_ = 3.05, m_aware_ = 3.43, p = .03); a similar pattern occurred for the parallel question focused on one’s own risk (m_unaware_ = 3.70, m_aware_ = 4.05, p = .07; Fig 3). Awareness was not related to the belief that e-cigarettes would cause lung damage.

Sub-groups of aware-unaware by user status are small and unstable but potentially instructive about the impact of the information circulating in the public environment. Due to the small numbers, we compared current users to non-users (both former users and never users) in sub-group analysis. For non-users, awareness of the story was associated with endorsement of the belief that using e-cigarettes was risky (m_unaware_ = 3.95, m_aware_ = 4.4, p = .06, Hedges’ g effect size = -.59; Table 4). There was no difference in endorsement between aware and unaware current users (m_unaware_ = 3.33, m_aware_ = 3.30, ns, effect size = .03).

**Table 4:** Mean belief endorsement about e-cigarettes among participants aware and unaware of the EVALI news story by user status and wording variation.

		Self-referent			Comparison		
**All participants**		**Aware of EVALI news (n = 135)**	**Unaware of EVALI news (n = 37)**	**Hedges’ g effect size**	**Aware of EVALI news (n = 132)**	**Unaware of EVALI news (n = 39)**	**Hedges’ g effect size**
	**Risky**	**4.05**	**3.70**^	**-.36**	**3.43**	3.05*	**-.35**
	**Causes lung damage**	4.02	3.70	-.34	3.46	3.30	-.14
	**Long-term health benefits**	2.76	2.77	.01	**2.59**	**3.02**^	**.32**
**Current users**		Aware (n = 43)	Unaware (n = 15)		Aware (n = 46)	Unaware (n = 11)	
	**Risky**	3.30	3.33	.03	3.09	3.27	.14
	**Causes lung damage**	3.53	3.33	-.22	3.26	3.45	.16
	**Long-term health benefits**	3.16	2.8	-.32	3.19	3.09	-.08
**Non-users (never and former users)**		Aware (n = 92)	Unaware (n = 22)		Aware (n = 86)	Unaware (n = 28)	
	**Risky**	**4.41**	**3.95**^	**-.59**	**3.61**	2.96**	**-.69**
	**Causes lung damage**	4.25	3.95	-.35	3.57	3.25	-.31
	**Long-term health benefits**	2.57	2.76	.12	**2.27**	3.00**	**.55**

Note: E-cigarette beliefs were measured on a scale of strongly disagree (coded as 1) to strongly agree (coded as 5).

^p < .1

*p < .05

**p < .01.

For these same subgroups, when a *comparison to cigarettes is primed*, unaware non-users exhibited less acceptance of e-cigarette risk compared to cigarettes than aware never users (m_unaware_ = 2.96, m_aware_ = 3.61, p < .01; Hedges’ g effect size = -.69; Table 4). For long-term health benefits of e-cigarettes compared to cigarettes, lower endorsement occurred among the aware compared to the unaware (m_unaware_ = 3.00, m_aware_ = 2.27, p < .01, Hedges’ g effect size = -.55). Story awareness among current users of e-cigarettes exhibited no differences or trends approaching significance for beliefs that e-cigarettes are riskier, cause lung damage, or have long-term health benefits *compared to regular cigarettes*.

## Discussion

Study two took place before EVALI had been officially named, and before confirmation of THC and vitamin E acetate as the likely causes of EVALI. Some news stories mention these as potential causes in the coverage of the “mysterious vaping illness.” In a public information environment that was working perfectly in service to public health at the time of study two, we would expect EVALI news to cause an increase in harm perceptions, an increase in the belief that e-cigarettes were risky and caused lung damage, and a decrease in the beliefs that e-cigarettes had long-term benefits. Our study did not find such neat and tidy results, leading us to examine why the information environment led to some of the expected and hoped for outcomes, but not all. This study did find that attitudes and beliefs about e-cigarettes changed after EVALI news became a well-known story. But rather than perceptions and beliefs moving systematically within the population, there were differences in how much the beliefs changed over time between e-cigarette never, former, and current users and when evaluating e-cigarettes on their own, or in comparison to cigarettes. There were no beliefs that changed significantly for all user groups *and* when referencing cigarettes and not.

From a public health perspective, we would hope that the EVALI news stories in the information environment would reach and move the most at-risk population, in this case, the current users of e-cigarettes. While harm perceptions about e-cigarettes increased overall after the EVALI coverage, the change among never users was small. Importantly, never users already had higher perceptions of harm, and the perceptions of harm among current users after EVALI are still lower than never users and former users before the news coverage. While overall harm perceptions increased for current users, their beliefs about lung damage, risk, and long-term health benefits of e-cigarettes did not change. The fact that there are differences between never, former, and current users is consistent with research demonstrating the role of involvement on message processing and attitude change [37]. Because current users have more of a personal interest in the safety of e-cigarettes than never users, they are likely more attentive to the information, and as a result are affected in different ways.

These data suggest that contrasting information processing motivations between groups with different experiences using e-cigarettes moderated the effects of a prominent news event on beliefs about the risks and benefits of vaping. That current users’ harm perceptions increased following the EVALI news, suggests that safety concerns are likely a key difference between the groups. For never users, the news of EVALI validated their previous behavior, whereas it forced current users to reconcile their behavior with information that it is harmful.

It is instructive to note that different beliefs changed when the *comparison to regular cigarettes* was primed. While it is expected that public information environment after EVALI would increase the beliefs about lung damage and risk, it is perplexing that those beliefs only increased when in comparison to regular cigarettes. This may be a result of the larger news narrative around e-cigarettes. The EVALI story and news concerning e-cigarettes in general has focused extensively on their popularity with young people [6], while e-cigarette industry marketing has focused on the benefits of e-cigarettes in comparison to cigarettes [38]. Thus, smokers are evaluating EVALI in light of the larger news context. EVALI may make e-cigarettes less appealing to use and have seemingly fewer benefits and more risks compared to cigarettes.

Changes in participant’s perceptions of e-cigarettes were associated with awareness of the coverage of this prominent news story, underscoring the importance of working to ensure that coverage is a scientifically accurate as possible. Changing beliefs and perceptions after news coverage is consistent with other news topics [21, 39–41]. It is particularly important to use these news events that capture the public’s attention to provide accurate and not misleading information. A morning consult poll conducted in mid-September indicated that 34% of adults believed that the lung disease deaths were related to marijuana and THC-containing vapes, while 58% percent said nicotine e-cigarettes such as Juul were to blame [42]. Given the evolving nature of the story in the time leading to study two, which news reports were seen by the participants may have been important in how beliefs and harm perceptions were changed. However individual news exposure and awareness of specific news stories were not measured in this study.

One of the limitations of this study is that it was not originally designed to capture changes in the beliefs about e-cigarettes in reaction to the public news environment. Therefore, we do not have data in study one about news consumption, exposure, or awareness, nor do we have information about whether users in either study used THC in their e-cigarettes in addition to tobacco. Cases of EVALI varied by state, and while awareness of the EVALI story was very high compared to other news stories, some of the differences in awareness may be due to location of the participants, which we did not measure. Because the first study’s aim was to capture beliefs about MRTPs among former and current smokers, we do not have e-cigarette users who have never used cigarettes in our sample. We did not anticipate conducting study two while we were planning and conducting study one. As such, we had not designed study one to allow for follow up with participants, the cross-sectional nature of these studies does not allow us to infer causality. We believe that even with these limitations, the data provide insight into how beliefs about tobacco products can change when there are major news events. We used mainstream news coverage about the mysterious vaping illness as an indicator of the public information environment. This was not intended to be a content analysis of mainstream or social media news sources, which could tell us which stories were circulating and influential. Instead, we can describe what beliefs and perceptions changed and did not change at the height of EVALI new coverage.

When the public information environment is dominated by news stories about the health and safety of tobacco products, it presents an opportunity to change beliefs that are frequently targeted by paid health campaigns. The EVALI story, like any other, appears subject to selective perception [43]. Our experiences, attitudes, and existing beliefs shape how we view and interpret news stories. A news story about a novel and complex issue like the safety of e-cigarettes is particularly likely to evoke motivated reasoning processes [44], particularly among e-cigarette users who have both a physical and emotional interest in the issue. Current users may have read the stories more carefully or followed the nuances of the evolving story whereas never users may not have followed the news as closely. However, as current users have a personal interest in whether e-cigarettes are safe, they are motivated to process information in such a way that allows them to maintain that belief rather than change their behavior.

## Supporting information

S1 Data (XLSX)

S2 Data (XLSX)
